# Supplementary material for: Roadmap of phase transitions in hafnia-based superlattice films
Source: Nat Commun. 2026 Mar 31;17:4676. doi: 10.1038/s41467-026-71265-7 (PMC13201757; doi:10.1038/s41467-026-71265-7)
Supplement: Supplementary file 1 — Supplementary Information [file 41467_2026_71265_MOESM1_ESM.pdf]

Supplementary Information for:

Roadmap of phase transitions in hafnia-based superlattice films

Wan-Rong Geng[1,2,†], Bo-Rui Wang[1,3,†], Yin-Lian Zhu[1,2,4], Si-Rui Zhang[3], Min Liao[3], Xiu-Liang Ma[1,2,5,6,*]

[1]Bay Area Center for Electron Microscopy, Songshan Lake Materials Laboratory, Dongguan, 523830, China.

[2]Dongguan Institute of Materials Science and Technology, Chinese Academy of Sciences, Dongguan, 523808, China.

[3]School of Advanced Materials and Nanotechnology, Xidian University, Xi'an 710071, China.

[4]School of Materials Science and Engineering, Hunan University of Science and Technology, Xiangtan, 411201, China.

[5]Institute of Physics, Chinese Academy of Sciences, Beijing, 100190, China.

[6]Quantum Science Center of Guangdong-HongKong-Macau Greater Bay Area (Guangdong), Shenzhen, 510290, China.

†Authors contributed equally to this work.

*Correspondence should be addressed to X. L. Ma (Email: xlma@iphy.ac.cn).

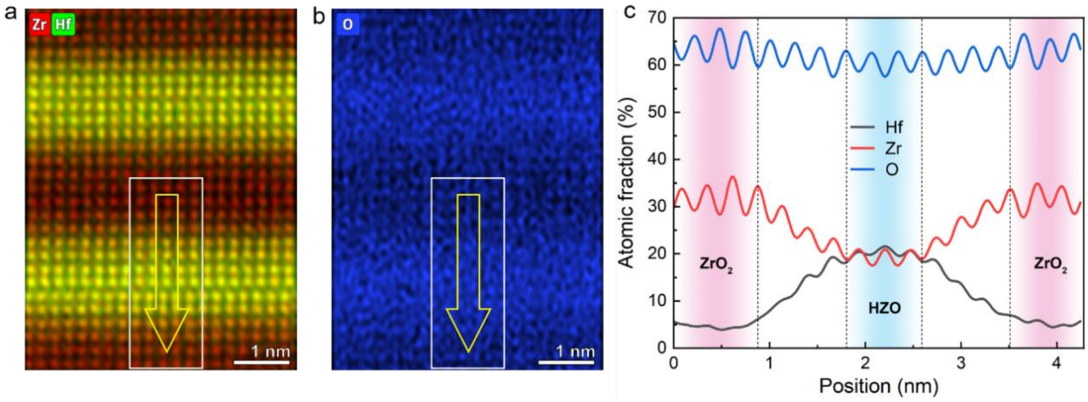

Supplementary Fig. 1| Elemental distribution in the superlattice film. a, Atomic-resolved EDS elemental map of Hf and Zr combined distribution. **b,** Atomic-resolved EDS elemental map of O distribution. **c,** Elemental spacing profiles of Hf, Zr, and O corresponding to the white rectangle in (a).

Supplementary Tab. 1| The quantitative EDS results of O and Zr in the ZrO_2 layer of the superlattice film.

Element	Atomic faction in ZrO_2	Atomic error in ZrO_2
O	65.4%	2.4%
Zr	27.8%	2.6%
Hf	6.8%	0.7%

Supplementary Tab. 2| The quantitative EDS results of O, Zr and Hf in the $Hf_{0.5}Zr_{0.5}O_2$ layer of the superlattice film.

Element	Atomic faction in $Hf_{0.5}Zr_{0.5}O_2$	Atomic error in $Hf_{0.5}Zr_{0.5}O_2$
O	65.7%	2.0%
Zr	18.9%	2.0%
Hf	15.4%	1.4%

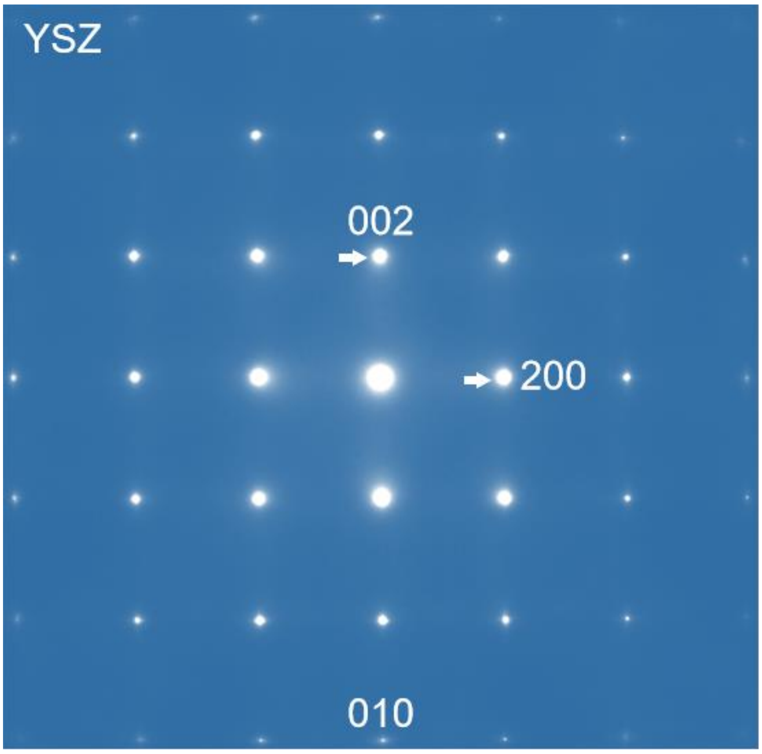

Supplementary Fig. 2| SAED pattern of the YSZ substrate projected along [010] zone axis.

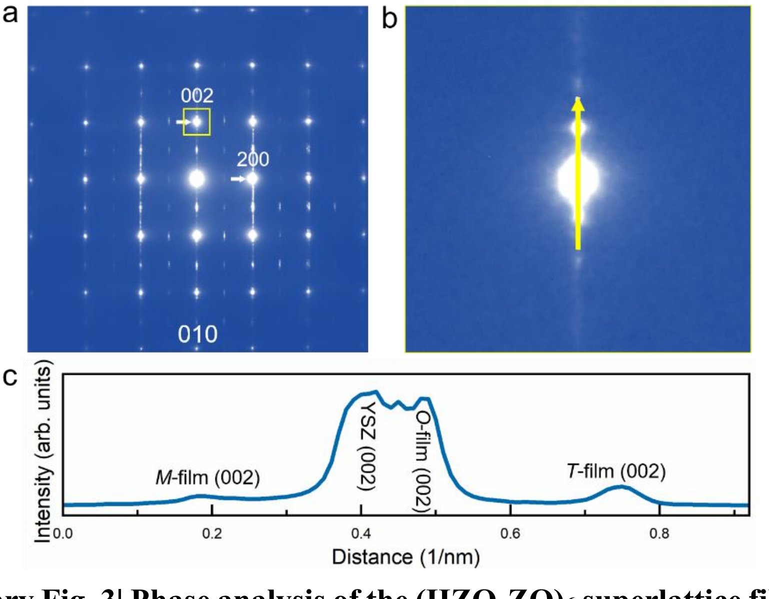

Supplementary Fig. 3| Phase analysis of the (HZO-ZO)$_6$ superlattice film. a, SAED pattern corresponding to Fig. 1f. **b,** Enlarged (002) diffraction corresponding to the yellow rectangle in (a). **c,** Intensity spacing profile corresponding to the yellow arrow in (b), suggesting the coexistence of *M*-phase, *O*-phase and *T*-phase in the hafnia-based superlattice film.

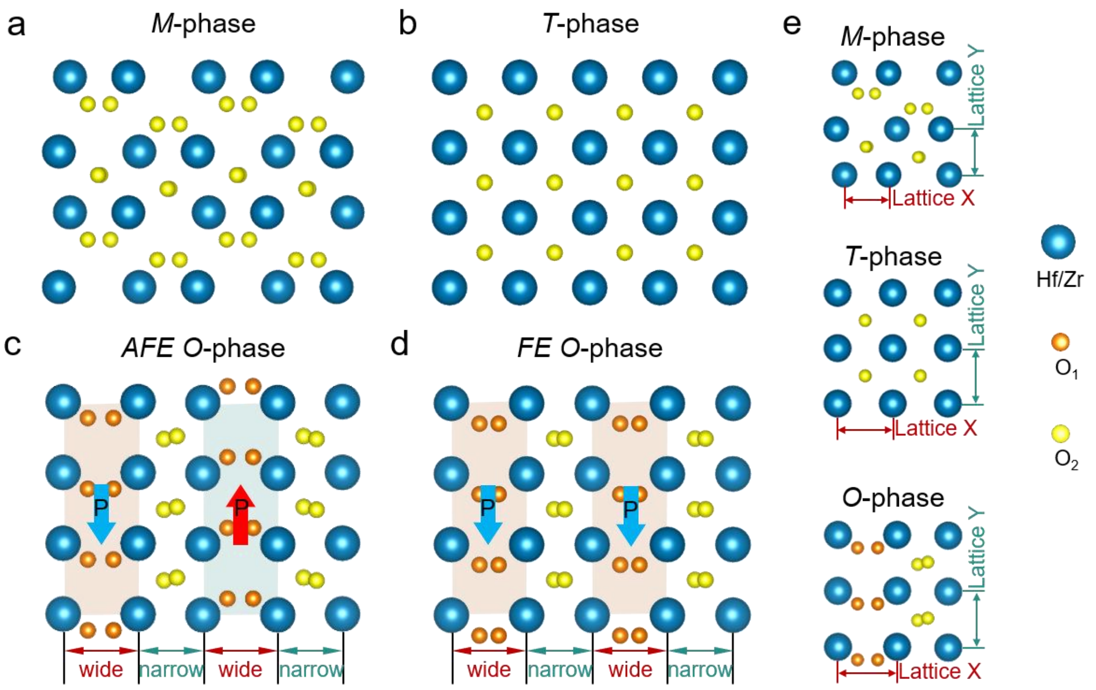

Supplementary Fig. 4| Multiple polymorphic phases in hafnia-based films. a, *M*-phase. **b,** *T*-phase. **c,** *AFE O*-phase. **d,** *FE O*-phase. The wide and narrow Hf/Zr sublattices are highlighted in the *AFE O*-phase and *FE O*-phase. **e,** Definition of the Lattice X and Lattice Y for different phases. Blue, orange and yellow balls denoting the Hf/Zr, O_1 and O_2 atomic columns, with the O_1 and O_2 representing the oxygen atoms in ferroelectric layer and spacer layer, respectively.

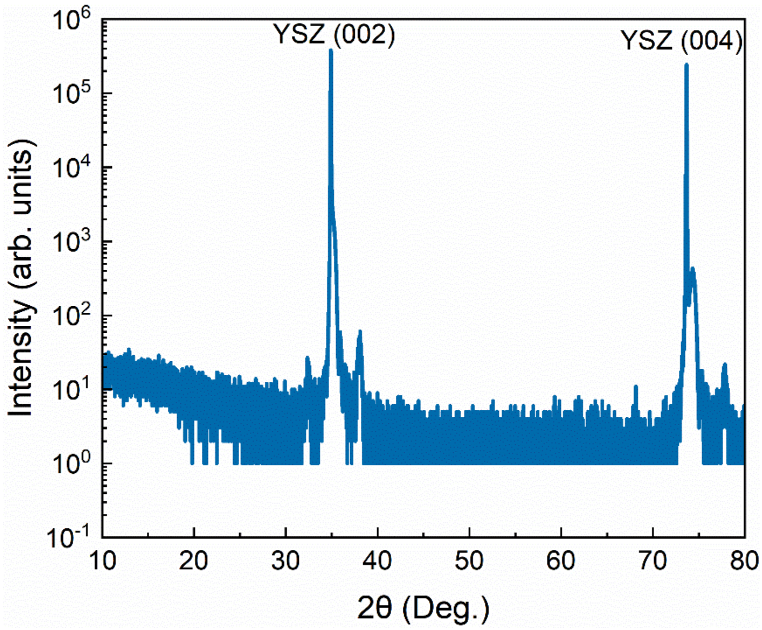

Supplementary Fig. 5| XRD θ-2θ scan from 10°-80° for the superlattice film.

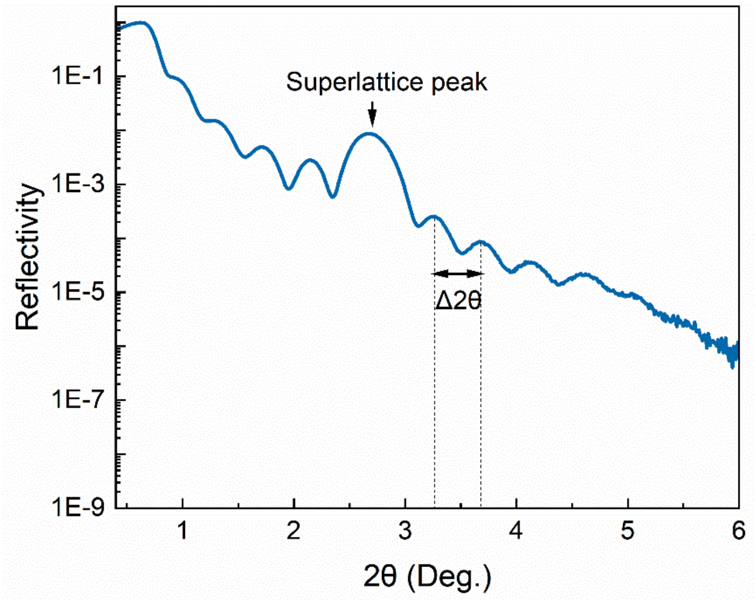

Supplementary Fig. 6| The XRR pattern of the superlattice film.

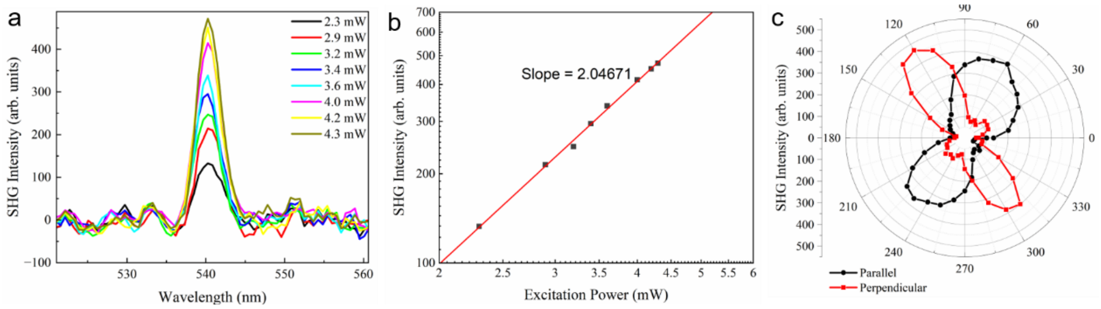

Supplementary Fig. 7| SHG characterizations of the superlattice film. a, Power-dependent SHG spectra. **b,** The excitation power dependence of SHG intensity with the coefficient fitted to 2.04671. **c,** Azimuth-dependent SHG signals of the superlattice film along parallel and perpendicular directions.

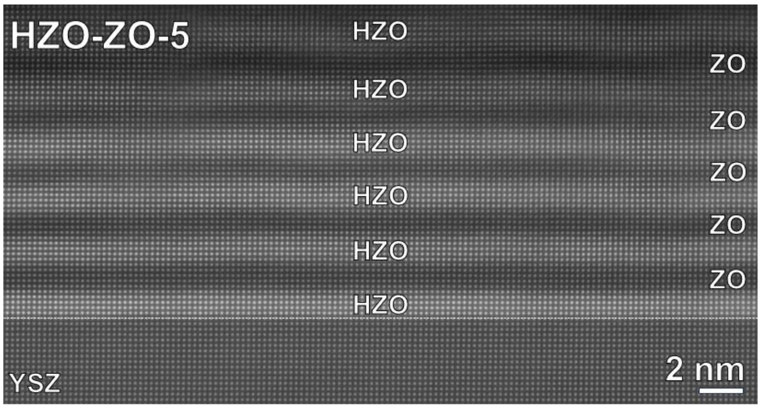

Supplementary Fig. 8| Atomic-resolved HAADF-STEM image of the (HZO-ZO-5)$_6$ superlattice film, with the average thickness of one (HZO-ZO) period being 5 unit-cells.

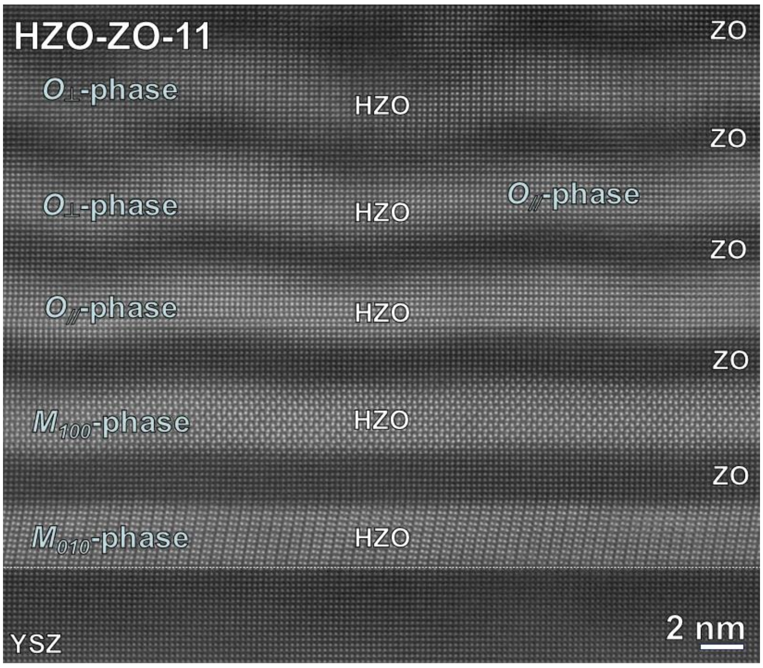

Supplementary Fig. 9| Atomic-resolved HAADF-STEM image of the (HZO-ZO-11)$_6$ superlattice film, with the average thickness of one (HZO-ZO) period being 11 unit-cells.

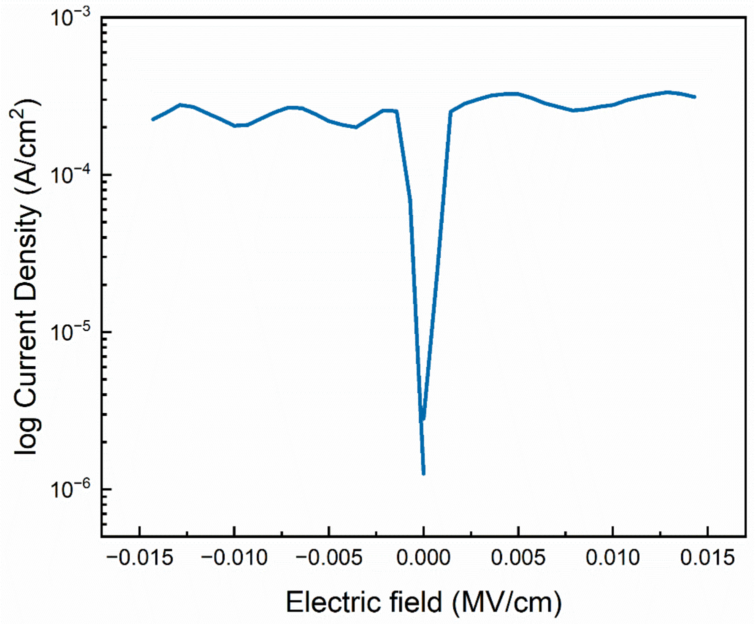

Supplementary Fig. 10| Leakage current curve as a function of electric field (J-E).

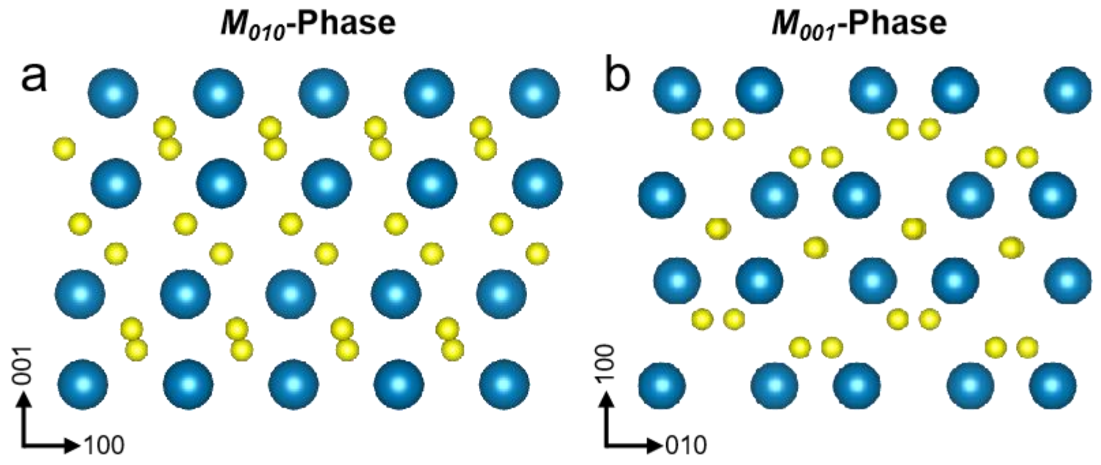

Supplementary Fig. 11| Schematics of the *M*-phase. a, *M*-phase projected along [010] zone axis. **b,** *M*-phase projected along [001] zone axis.

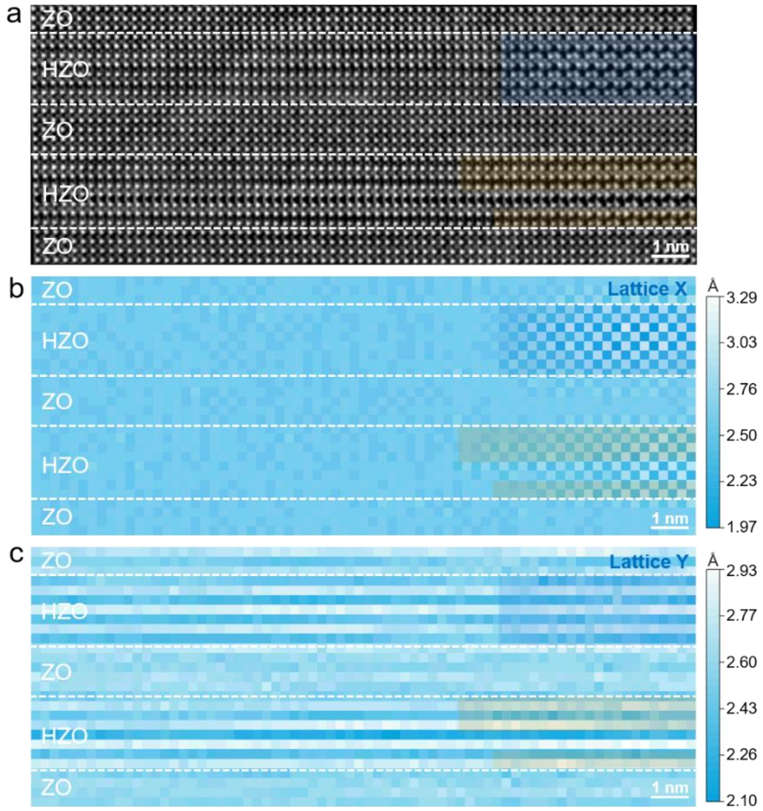

Supplementary Fig. 12| Lattice distortion of the *M_{001}'*-state and *M_{001}*-phase. a, Atomic-resolved iDPC-STEM image. **b,** In-plane lattice constant (Lattice X) map. **c,** Out-of-plane lattice constant (Lattice Y). Blue mask and yellow mask denoting the *M_{001}*-phase and *M_{001}'*-state, respectively.

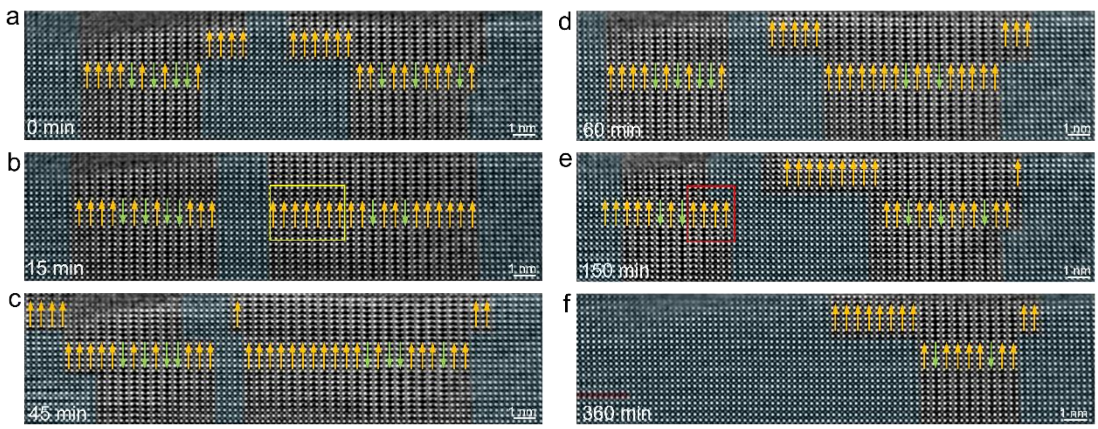

Supplementary Fig. 13| Reversible phase transition between *T*-phase and *O*-phase. a-f, Atomic-scale HAADF-STEM images after different irradiation durations: a, 0 min; b, 15 min; c, 45 min; d, 60 min; e, 150 min; f, 360 min, with the *T*-phase highlighted by blue mask and horizontal *O*-phase highlighted by red mask.

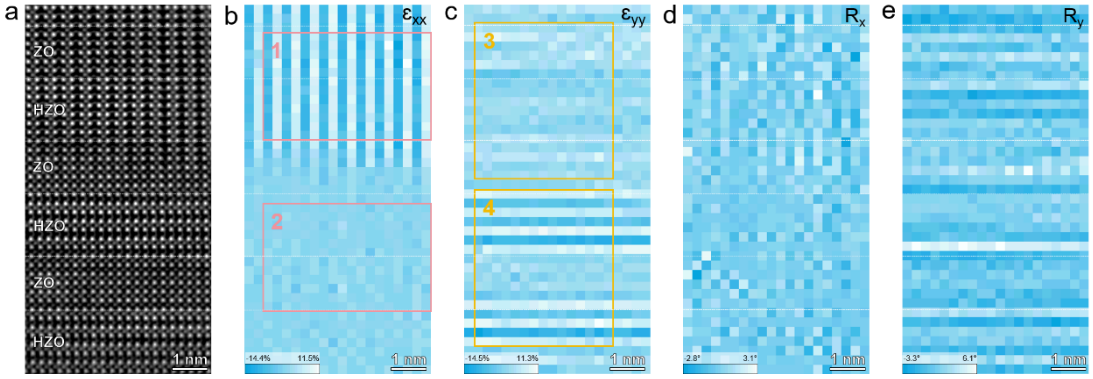

Supplementary Fig. 14| Detailed strain distribution for the *O*-phase. a, Atomic-resolved iDPC-STEM image including both the $O_{\perp}$-phase and $O_{//}$-phase. **b,** In-plane normal strain (ε_{xx}) map. **c,** Out-of-plane normal strain (ε_{yy}) map. **d,** In-plane lattice rotation (R_x) map. **e,** Out-of-plane lattice rotation (R_y) map.

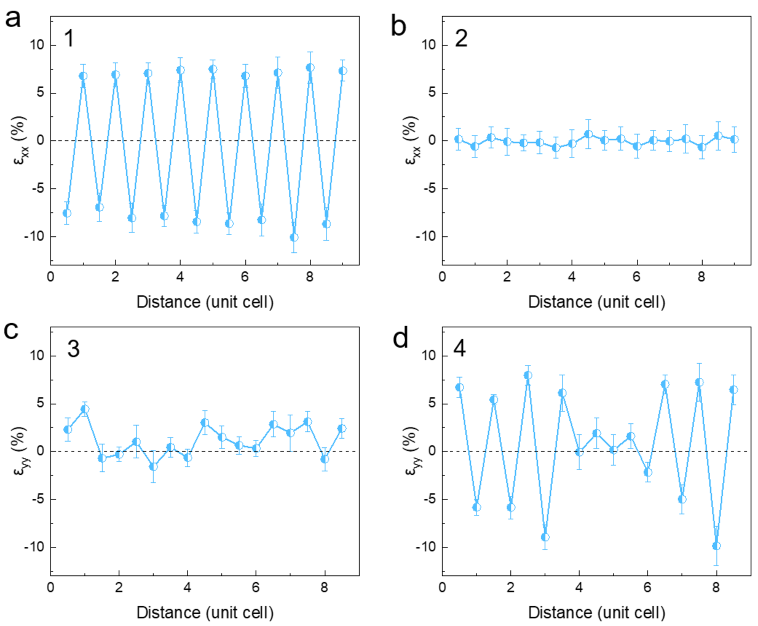

Supplementary Fig. 15| Quantitative strain analysis of Supplementary Fig. 14. a, The ε_{xx} spacing profile for the region 1 along in-plane direction. **b,** The ε_{xx} spacing profile for the region 2 along in-plane direction. **c,** The ε_{yy} spacing profile for the region 3 along out-of-plane direction. **d,** The ε_{yy} spacing profile for the region 4 along out-of-plane direction.

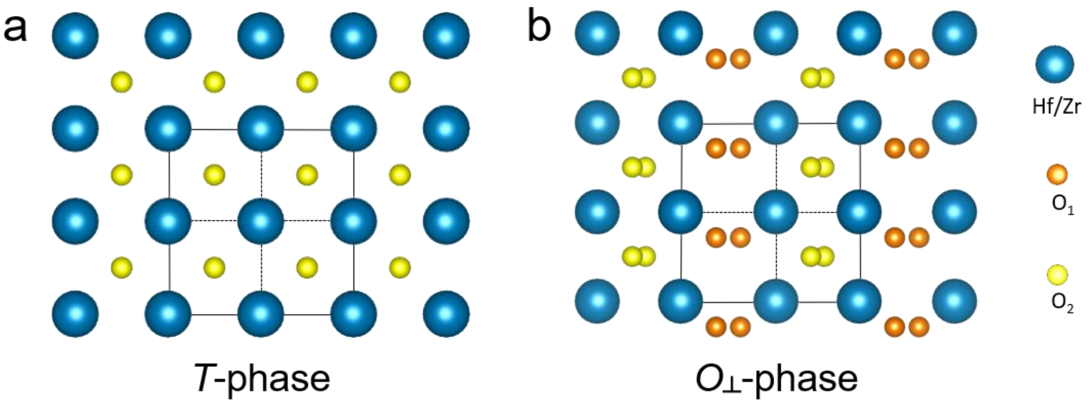

Supplementary Fig. 16| Unit cell schematics of *T*-phase (a) and *O*⊥-phase (b).

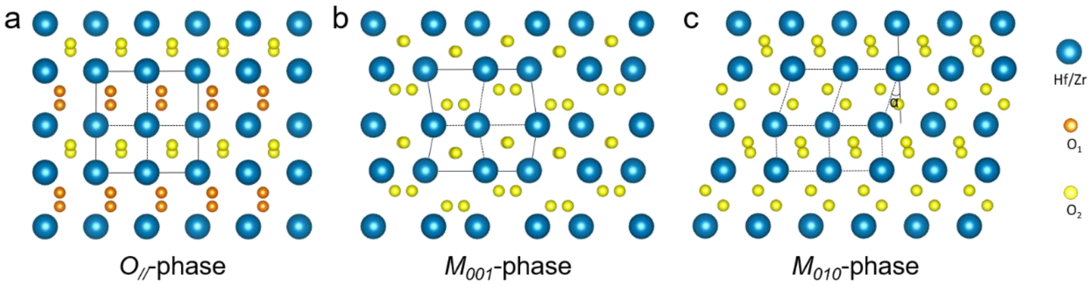

Supplementary Fig. 17| Unit cell schematics of *O*//-phase (a), *M*$_{001}$-phase (b) and

***M_{010}*-phase (c).**

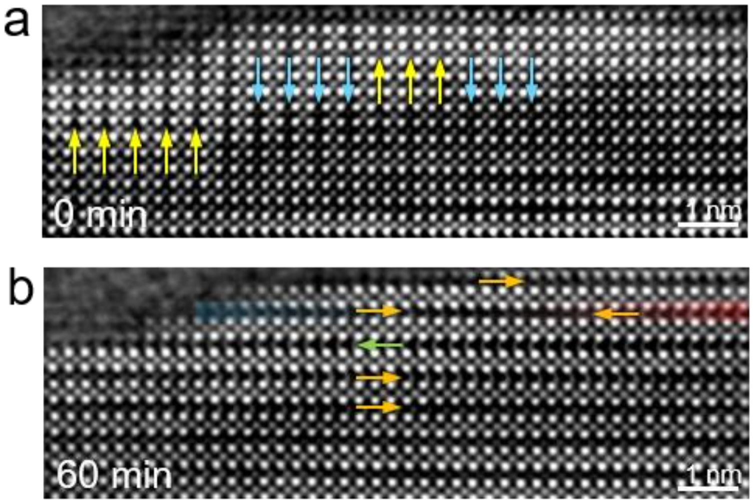

Supplementary Fig. 18| Ferroelastic switching of *O*-phase under electron beam irradiation. a, The *FE-O* phase with vertical polarization. **b,** The *FE-O* phase with horizontal polarization.
